# Comparative Review of Postoperative Analgesic Use After Total Hip Replacement: Opioids Versus Non-opioids

**DOI:** 10.7759/cureus.68237

**Published:** 2024-08-30

**Authors:** Gursimran Singh, Nareshkumar Dhaniwala, Vivek H Jadawala, Ankur Salwan, Nitish Batra

**Affiliations:** 1 Orthopaedics, Jawaharlal Nehru Medical College, Datta Meghe Institute of Higher Education and Research, Wardha, IND; 2 Medicine, Jawaharlal Nehru Medical College, Datta Meghe Institute of Higher Education and Research, Wardha, IND

**Keywords:** analgesic efficacy, patient recovery, pain control, regional anesthesia, acetaminophen, nsaids, non-opioid analgesics, opioids, postoperative pain management, total hip replacement

## Abstract

Total hip replacement (THR) is a common surgical procedure aimed at alleviating pain and improving function in patients with hip joint pathology. Effective postoperative pain management is crucial for patient recovery, satisfaction, and overall outcomes. This narrative review examines the comparative efficacy, safety, and implications of using opioids versus non-opioid analgesics in managing postoperative pain following THR. Opioids, while effective for severe pain, pose significant risks such as addiction, tolerance, and adverse effects. Non-opioid analgesics, including non-steroidal anti-inflammatory drugs (NSAIDs), acetaminophen, and regional anesthesia techniques, offer alternatives with potentially fewer side effects. This review synthesizes current evidence from clinical trials, observational studies, and expert guidelines to provide a comprehensive understanding of the benefits and drawbacks of each analgesic approach. The goal is to inform clinical decision-making and optimize pain management strategies for THR patients, balancing efficacy and safety.

## Introduction and background

Total hip replacement (THR) surgery is a common and effective procedure intended to alleviate pain and improve function in patients with hip joint disorders. However, THR carries significant risks after surgery; therefore, there must exist effective methods to manage pain in order to get better recovery outcomes for people [1]. Generally, postoperative pain management has relied heavily on opioids because of their powerful analgesic effect. However, recent developments in opioid use have highlighted the need to find other ways to manage chronic pains without causing any harm to our bodies or our brains [2]. The inclusion of non-opiate analgesics such as non-steroidal anti-inflammatory drugs (NSAIDs), acetaminophen, and regional anesthetic practices has made them possible substitutes for or adjuncts to opioid therapies [3]. Such alternatives strive to deliver effective pain relief while reducing adverse effects associated with opioids. The comparative effectiveness, safety, and overall impact on patient outcomes in THR between opioid and non-opioid analgesics are still researchable and arguable issues [4].

Recent years have witnessed a growing interest in non-opioid analgesics, including NSAIDs, acetaminophen, and regional anesthesia techniques. These alternatives aim to provide effective pain control while minimizing the risk of opioid-related complications. Comparative studies have increasingly focused on the efficacy, safety, and patient outcomes associated with opioid versus non-opioid regimens in the context of THR [2]. This review aims to synthesize current evidence on the comparative effectiveness of these analgesic strategies, with an emphasis on optimizing pain management while addressing the broader public health implications of opioid use. This narrative review aims to provide a comprehensive overview of the current evidence on the use of opioids and non-opioids for postoperative pain management following THR. By examining the benefits, risks, and outcomes associated with each approach, this review seeks to inform clinical practice and guide decision-making in optimizing pain management for THR patients.

## Review

Search methodology

A comprehensive literature search was conducted to identify studies comparing postoperative analgesic use of opioids and non-opioids following THR. Searching databases such as PubMed, Embase, and Cochrane Library included works from January 2000 through June 2024. The keywords were "total hip replacement," "postoperative analgesia," "opioids," and "non-opioids." To be included, randomized controlled trials, cohort studies, and systematic reviews had to compare adults undergoing THR in terms of effectiveness, safety, and outcomes of opioid versus non-opioid medications. Inclusion criteria consisted mainly of randomized controlled trials, cohort studies, and systematic reviews that compared the efficacy, safety, and results of opioid versus non-opioid pain relievers after THR among adults. It also required studies that provided clear data regarding pain management outcomes, e.g., pain scores, side effects, and patient satisfaction. Excluded were those written in other languages apart from English, case reports, systematic reviews without primary studies, pediatric-focused studies, or frameworks discussing replacements following traumatically broken hips or cancerous cases. The only papers looked at were peer-reviewed articles in order to guarantee the quality assurance and reliability of the information studied: postoperative pain management.

Pain mechanisms after THR

Surgical Trauma

Surgical insults resulting from THR are an unavoidable outcome due to their invasive nature, which entails extensive dissection, skeletal resection, and even placing implants into the body. Several factors contribute to this kind of trauma, including the surgical approach, which could be posterior, lateral, or anterior [3]. The trauma to soft tissues, bones, and nerves during the procedure triggers the release of pro-inflammatory cytokines and chemokines, leading to peripheral sensitization and subsequent central sensitization, where the nervous system becomes hyper-responsive to pain stimuli. This heightened pain perception complicates recovery, making effective pain management crucial. In this context, the choice between opioids and non-opioids as postoperative analgesics becomes significant, as opioids, while potent, carry risks of dependency and adverse effects. In contrast, non-opioids, including NSAIDs and acetaminophen, offer pain relief with a lower side effect profile but may be less effective in severe pain cases. The balance between managing surgical trauma-induced pain and minimizing adverse outcomes is central to optimizing postoperative care [5].

Inflammatory Response

The inflammatory response plays a significant role in the pain mechanisms subsequent to THR. After surgery, tissue injury leads to the release of pro-inflammatory cytokines such as tumor necrosis factor-alpha (TNF-α), interleukin-1 (IL-1), and interleukin-6 (IL-6). These cytokines are critical mediators in the inflammatory cascade. They attract immune cells such as neutrophils and macrophages to the injury site, further amplifying the inflammatory response [6]. This response facilitates tissue repair but also sensitizes nociceptors, leading to an increased pain perception, which is referred to as hyperalgesia. Furthermore, the release of prostaglandins and other inflammatory mediators exacerbates pain by lowering activation thresholds for sensory neurons. If left unchecked, progressive inflammatory response can result in both acute and chronic pain, promoting postoperative discomfort and influencing recovery processes. It is essential to develop specific interventions aimed at relieving postoperative pain and improving patient outcomes to understand the complex interaction between inflammation and pain after THR [6].

Neuropathic Components

Following THR, pain mechanisms may involve neuropathic components, particularly if nerve damage or irritation occurs during the surgery. This nerve injury can lead to neuropathic pain, characterized by abnormal pain sensations, such as burning, tingling, or shooting pain, and may complicate the overall pain management process following the procedure [2]. Neuropathic pain appears as burning, tingling, or shooting sensations that sometimes occur because of injury to nerves such as the sciatic or femoral nerve, which could either happen during the procedure itself or postsurgical inflammation. The existence of these neuropathic components complicates the control of pain. It may require different therapeutic approaches from those used with nociceptive pain, which usually results from tissue damage or inflammation. Studies indicate that even when the surgical wound has healed, neuropathic pain can still be present, thereby contributing to chronic pain and reduction in quality of life for patients. In controlling post-THR neuropathic pain, it is essential since this type does not respond well to conventional analgesic drugs but needs medications such as gabapentinoids or tricyclic antidepressants [7].

Psychosocial Factors

The experience of pain following THR can make one suffer from the ways that psychosocial factors are involved. That means they can increase the severity and persistence of postoperative pain experienced by patients who have undergone THR. Psychological elements such as anxiety, depression, and catastrophizing are strongly correlated with increased pain perception after surgery. Patients with higher levels of preoperative anxiety or depressive symptoms tend to report more incredible postoperative pain, possibly due to increased sensitivity to pain stimuli or a negative interpretation of sensations related to this condition [5]. To sum up, psychosocial factors can have a significant impact on postoperative pain following THR. For instance, it is not uncommon for people who lack any form of support network to suffer more from loneliness and stress, which amplify the sensations of hurt that they feel throughout their lives. Furthermore, cultural attitudes towards suffering and personal coping strategies also affect how we perceive hurtful occurrences. It has been argued that cognitive-behavioral therapy could help minimize these issues, leading to reduced levels of discomfort associated with THR surgery while improving overall rehabilitation outcomes [8].

Goals of pain management

Pain Relief

The primary goal is to alleviate pain to a manageable level, enabling the patient to rest, recover, and engage in necessary postoperative activities such as physical therapy. Effective pain relief improves patient satisfaction and overall quality of life during the recovery period [9].

Enhancing Mobility and Function

Adequate pain control facilitates early mobilization, which is critical for preventing complications such as deep vein thrombosis, pulmonary embolism, and muscle atrophy. Pain management should support the patient's ability to participate in rehabilitation exercises and activities of daily living [10].

Minimizing Side Effects

The choice of analgesics should consider the balance between efficacy and potential side effects. Opioids, while effective, can cause adverse effects such as nausea, constipation, and sedation. Non-opioid analgesics, including NSAIDs and acetaminophen, are often used to minimize these side effects while providing effective pain relief [11].

Preventing Chronic Pain

Proper management of acute postoperative pain can reduce the risk of developing chronic pain. Multimodal analgesia, combining different classes of pain medications, can address various pain pathways and reduce the likelihood of long-term pain issues [12].

Individualized Patient Care

Pain management strategies should be tailored to the individual needs of each patient, taking into account their medical history, pain tolerance, and any pre-existing conditions. Personalized pain management plans can enhance recovery outcomes and patient satisfaction [13].

Opioid analgesics

Opioid analgesics have long been a cornerstone in managing postoperative pain, particularly after major surgeries such as THR. These potent medications are effective at reducing severe pain by acting on the central nervous system (CNS) to block pain signals. However, their use is associated with numerous benefits and drawbacks that need careful consideration [9].

Mechanism of Action

Opioids cause pain relief by fitting into specific receptors (mu, kappa, and delta) located in your brain and spine. Apoplexy stops because information concerning the injury is prevented from reaching the cortex, and a person's mood towards suffering is modified with them. Frequently applied ones in the surgery recovery period are morphine, oxycodone, hydromorphone, and fentanyl [14].

Efficacy

Opioids are highly effective analgesic agents for the management of acute postoperative pain. Research shows that they can greatly decrease levels of discomfort and alleviate suffering during the early moments after an operation. Due to their rapid-onset working elements in terms of time, it is far more beneficial for pain when simple medications such as paracetamol are not sufficient enough to deal with [15].

Administration Routes

The routes by which opioids can be given include orally, intravenously, intramuscularly, and even epidurally. The choice of administration is based on the patient's state, how bad it hurts for them, and the clinical setting where such medication is provided. Administering through IV serves faster pain relief, making it ideal for the immediate postsurgical phase. In contrast, most other ongoing management for pains following minor surgeries uses oral opioids [16].

Side Effects and Risks

Even though opioids are very effective, they have numerous side effects and complications. Nausea, vomiting, constipation, and sedation are some of the common side effects. Serious effects consist of respiratory depression, which can prove to be fatal, particularly with new users on opioids or when they are used in high amounts. Chronic usage of opioids also results in reliance, tolerance, and addiction [17].

Opioid-Sparing Strategies

In light of the dangers that come with taking opioids, there is a progressively increasing importance on avoiding these drugs after surgery. They involve the use of multimodal analgesia whereby narcotics are combined with non-narcotic painkillers, as well as adjunctive medicines, all aimed at reinforcing their ability to relieve pain while reducing their usage. It is also possible to use regional anesthetic methods such as nerve blocks to minimize times when general anesthetics have to be used [18].

Comparison With Non-opioid Analgesics

Non-opioid analgesics, such as NSAIDs, acetaminophen, and gabapentinoids, offer an alternative to opioids for postoperative pain management. While these medications may be less potent than opioids, they carry a lower risk of severe side effects and addiction. The comparative effectiveness and safety of opioids versus non-opioids are critical considerations in developing postoperative pain management protocols for patients undergoing THR [11].

Non-opioid analgesics

Non-opioid analgesics are increasingly being considered as viable alternatives to opioids for managing postoperative pain following THR. These analgesics offer effective pain relief with a lower risk of side effects and dependency. The main categories of non-opioid analgesics include NSAIDs, acetaminophen, and adjuvant medications such as gabapentinoids and corticosteroids. Each of these has unique mechanisms of action and benefits in the context of postoperative pain management [19].

NSAIDs

NSAIDs are commonly used to reduce inflammation and alleviate pain. They work by inhibiting the cyclooxygenase (COX) enzymes, COX-1 and COX-2, which are involved in the synthesis of prostaglandins that mediate inflammation and pain. The use of NSAIDs in postoperative care can help manage moderate pain and reduce the need for opioids [20].

Acetaminophen

Acetaminophen, also known as paracetamol, is another cornerstone of non-opioid analgesia. It is effective for mild to moderate pain relief and can be used with other analgesics to enhance pain control [21].

Adjuvant Medications

Adjuvant medications such as gabapentinoids (gabapentin and pregabalin) and corticosteroids can also be part of a multimodal analgesia approach. These medications do not directly relieve pain but can enhance the effects of primary analgesics and manage specific symptoms such as neuropathic pain [22].

Efficacy and Safety

The efficacy of non-opioid analgesics in postoperative pain management has been well-documented. Studies have shown that multimodal analgesia, combining non-opioid analgesics with opioids or other pain relief methods, can provide superior pain control compared to opioids alone. This approach can lead to reduced opioid consumption, fewer side effects, and improved patient outcomes [23].

Comparative analysis

Direct Comparisons Between Opioids and Non-opioids

When analyzing postoperative analgesic use after THR, it is crucial to compare the efficacy of opioids and non-opioids in managing pain. Opioids, such as morphine, oxycodone, and hydrocodone, are often prescribed due to their potent analgesic effects. Non-opioids, including acetaminophen, NSAIDs (like ibuprofen and celecoxib), and COX-2 inhibitors, provide alternatives that may reduce pain with fewer risks of dependency. Studies consistently show that while opioids are highly effective for severe pain, non-opioids can be equally effective for moderate pain with a better safety profile [9].

Side Effect Profiles

Opioids: Opioids possess strong analgesic properties that often find their application both in the management of acute and chronic pain. However, the multitude of side effects drastically limits their utilization, thus affecting the quality of life for such patients and adherence to treatment regimens. Some common side effects include constipation, nausea, vomiting, dizziness, and sedation. Also, opioids are able to cause a high level of dependence, leading to addiction, which is a major issue with long-term therapy. Respiratory depression is another adverse effect, which can be very severe and sometimes fatal, especially when dealing with overdoses or when mixed with other CNS depressants. Chronic use of opioids can also lead to an increase in patients' sensitivity to pain, referred to as opioid-induced hyperalgesia. Other long-term consequences that accompany opioid therapy include cognitive dysfunction and endocrine system disorders such as hypogonadism. Such medications need close attention and the lowest possible doses to prevent dangers while granting adequate analgesia [24].

Non-opioids: Non-opioid analgesics are a class of drugs frequently used to manage pain and inflammation, providing an alternative for managing pain and inflammation with a different mechanism of action and generally a better side effect profile than opioids. Commonly used non-opioids include analgesics such as acetaminophen (paracetamol) and NSAIDs such as ibuprofen, aspirin, and naproxen. Acetaminophen is extensively recognized as effective in relieving mild to moderate pains and lowering fever levels without causing gastrointestinal (GI) complications [9]. However, when taken in excessive amounts, it can cause damage to the liver, particularly among those people who have existing liver conditions. On the other hand, NSAIDs are useful in alleviating pain and inflammation, but they are associated with an increased likelihood of developing GI complications such as gastritis ulcers and bleeding, especially when they have been administered over a long period. Furthermore, NSAIDs may escalate the chances of heart attacks or strokes, particularly among patients who already have heart diseases. Additionally, renal toxicity is also an issue, especially among people with compromised kidneys or who have used these medications for extended intervals. Notwithstanding these potential hazards, non-opioids are generally deemed safer than opioids, particularly concerning chronic pain management, because they have lesser tendencies towards dependence or misuse [11].

Risk of dependency and addiction

One of the most significant concerns with opioid use is the risk of dependency and addiction. Prolonged opioid use can lead to tolerance, requiring higher doses for the same pain relief, and eventually physical dependence. This risk is a critical consideration for both patients and healthcare providers [25]. In contrast, non-opioids present a much lower risk of dependency and addiction. Their use is often preferred for managing pain where opioids are not strictly necessary. By minimizing exposure to opioids, patients can avoid the potential for developing opioid use disorder, a growing concern in postoperative care [26].

Discussion

Summary of Findings

We learned about the effectiveness of opioids and non-opioids for postoperative analgesia after THR. Multiple studies were analyzed in various areas, such as pain management, patient satisfaction, adverse effects, and functional recovery associated with each analgesic approach. The results revealed that both opioid and non-opioid analgesics can effectively manage postoperative pain but have entirely different profiles of side effects and impact on patient outcomes. Opioids were generally associated with more adverse effects such as nausea, vomiting, constipation, and the possibility of becoming dependent, while few NSAID types, such as aspirin or paracetamol, have been shown to keep these cases under control [27].

Clinical Implications

The decision regarding whether to use opioids or non-opioids for postoperative pain management after THR has significant clinical implications. The results show that personalized approaches are necessary for relieving pain and include considerations such as a person's ability to handle pain, susceptibility to side effects, and lack of prior drug abuse [28]. It is essential for clinicians to look at the advantages of effective pain control vis-a-vis the disadvantages related to opioid side effects and addiction. Non-opioids may be better for many patients because they have fewer adverse reactions and work just as well when it comes to alleviating discomfort. Further still, multimodal analgesia, which combines both kinds of medications, would probably provide an ideal amalgam between proper pain relief on the one hand and a lower incidence rate of undesirable outcomes on the other [29].

Limitations of the current review

This review has some limitations that ought to be recognized first. The different types of study designs, patient populations, and the variety of analgesic protocols used in the research can affect its applicability. Besides, there is the likelihood of publication bias in terms of available studies on account of relying on published literature, as some of them with negative or uncertain results may not be found. Thirdly, because each study measures pain and adverse effects differently, it becomes difficult to compare them directly. Most importantly, opioid-related long-term outcomes such as dependence and quality of life were not sufficiently addressed within the included studies, hence suggesting that additional investigations are essential in this domain.

## Conclusions

The recovery of patients with THR depends on effective management of postoperative pain. While opioids have very high potency, they also come with numerous risks and side effects. Meanwhile, non-opioid analgesics serve as a better choice given their similar strength in managing postoperative pain. Future research should be geared towards coming up with pain management protocols that are effective yet safe by incorporating multimodal analgesic approaches, which will improve patient outcomes and lessen reliance on opioids.
